# Problematische Nutzung digitaler Medien und Gesundheitskompetenz von Schülerinnen und Schülern in Deutschland. Befunde der HBSC-Studie 2022

**DOI:** 10.1007/s00103-025-04008-6

**Published:** 2025-02-18

**Authors:** Kevin Dadaczynski, Anne Kaman, Ulrike Ravens-Sieberer, Saskia M. Fischer, Ludwig Bilz, Saskia Sendatzki, Ronja M. Helmchen, Katharina Rathmann, Matthias Richter, HBSC-Studienverbund Deutschland

**Affiliations:** 1 Fachbereich Gesundheitswissenschaften, Hochschule Fulda, Leipziger Straße 123, 36037 Fulda, Deutschland; 2 Zentrum für Angewandte Gesundheitswissenschaften, Leuphana Universität Lüneburg, Lüneburg, Deutschland; 3 Zentrum für Psychosoziale Medizin, Klinik für Kinder- und Jugendpsychiatrie, -psychotherapie und -psychosomatik, Forschungssektion Child Public Health, Universitätsklinikum Hamburg-Eppendorf, Hamburg, Deutschland; 4 Fakultät für Humanwissenschaften, Brandenburgische Technische Universität Cottbus-Senftenberg, Senftenberg, Deutschland; 5 Fachbereich Sozialwesen, Hochschule RheinMain, Wiesbaden, Deutschland; 6 School of Medicine and Health, Lehrstuhl Social Determinants of Health, Technische Universität München, München, Deutschland; 7 Public Health Zentrum Fulda (PHZF), Hochschule Fulda, Fulda, Deutschland

**Keywords:** Soziale Medien, Digitale Spiele, Gesundheitsinformationen, Kinder und Jugendliche, Prävalenz, Social media, Games, Health information, Children and adolescents, Prevalence

## Abstract

**Hintergrund:**

Der digitale Alltag von Kindern und Jugendlichen ist durch eine hohe Nutzung sozialer Medien und digitaler Spiele geprägt. Bisherige Forschung konnte gesundheitsabträgliche Konsequenzen einer problematischen Mediennutzung aufzeigen. Hingegen wurden Zusammenhänge mit der Gesundheitskompetenz kaum untersucht, obgleich Rahmenmodelle Gesundheitskompetenz als Determinante des Gesundheitsverhaltens nahelegen.

**Methoden:**

Dieser Beitrag greift auf Daten der repräsentativen Studie „Health Behaviour in School-aged Children“ (HBSC) in Deutschland aus dem Jahr 2022 mit *n* = 6475 Schülerinnen und Schülern zurück. Es wurden Zusammenhänge zwischen der problematischen Nutzung von sozialen Medien und der Videospielintensität sowie der Gesundheitskompetenz unter Berücksichtigung soziodemografischer und -ökonomischer Merkmale bivariat und multivariat untersucht.

**Ergebnisse:**

Die Prävalenz der problematischen Nutzung sozialer Medien betrug 11,1 % und etwa ein Drittel gab an, mehr als 9 h pro Woche mit digitalen Spielen zu verbringen. Eine geringe Gesundheitskompetenz stand mit beiden Formen der Mediennutzung in signifikantem Zusammenhang. Zudem war die problematische Nutzung sozialer Medien mit einer weiblichen und genderdiversen Geschlechtszugehörigkeit, dem Alter von 13 Jahren, dem Vorliegen eines Migrationshintergrunds und der Zugehörigkeit zu einer anderen Schulform als das Gymnasium verbunden. Eine hohe Spielintensität war mit einer männlichen und genderdiversen Geschlechtszuordnung, der Zugehörigkeit zu den Altersgruppen der 13- und 15-Jährigen und einem geringen familiären Wohlstand assoziiert.

**Diskussion:**

Die Ergebnisse liefern nicht nur Hinweise für die Identifikation von Heranwachsenden mit besonderem Präventionsbedarf, sondern betonen auch die Relevanz, die der Gesundheitskompetenz für das Mediennutzungsverhalten zukommt. Exemplarische Empfehlungen werden unter Rückgriff auf die internationale Literatur aufgegriffen.

## Abstract (en)

**Background:**

The digital life of children and young people is characterised by frequent use of social media and digital games. Previous research has demonstrated detrimental health effects of problematic media use. However, links with health literacy (HL) have received little attention, although frameworks suggest health literacy to be a determinant of health behaviour.

**Methods:**

This paper draws on data from the representative Health Behaviour in School-Aged Children (HBSC) Study in Germany from 2022 with *n* = 6475 pupils. Associations between problematic social media use or gaming intensity with health literacy and socio-demographic and socio-economic characteristics were examined using bivariate and binary-logistic regression analyses.

**Results:**

The prevalence of problematic social media use was 11.1% and approximately one third reported to spend more than nine hours a week playing digital games. Low health literacy was found to be significantly associated with both forms of media use. In addition, problematic social media use was associated with a female and diverse gender, an age of 13 years, a migrant background and belonging to a type of school other than grammar/high school. High gaming intensity was associated with a male and diverse gender, belonging to the 13- and 15-years age group and low family affluence.

**Discussion:**

The results not only provide guidance for identifying adolescents with a high prevention need but also emphasize the relevance of health literacy for media use behaviour. Exemplary recommendations are taken up with reference to the international literature.

## Einleitung

Die technologische Entwicklung der vergangenen Jahrzehnte hat dazu geführt, dass digitale Endgeräte und deren Anwendungen insbesondere für Kinder und Jugendliche zum selbstverständlichen Bestandteil des Alltags gehören [1]. Dabei stehen vor allem soziale Medien im Vordergrund des Nutzungsinteresses, die nach einer aktuellen repräsentativen Studie von 10- bis 17-Jährigen wochentags durchschnittlich 150 min und am Wochenende 224 min genutzt werden [2]. Für digitale Spiele als weitere Form der Mediennutzung ergeben sich ebenfalls hohe tägliche Nutzungszeiten, die in Abhängigkeit von Studie und Zeitraum (Wochentag versus Wochenende) zwischen 92 min und 168 min liegen [1, 2]. Neben Geschlechtsunterschieden (häufigere Spielnutzung bei Jungen und häufigere Nutzung sozialer Medien bei Mädchen) zeigt sich für digitale Spiele eine weniger häufige Nutzung bei Heranwachsenden aus dem Gymnasium [1–3].

Im Hinblick auf ihre gesundheitliche Bedeutung werden digitale Medien kontrovers diskutiert. Dabei reicht die Spannweite von der Gesundheitskommunikation mit gesundheitsförderlichen Wirkungsannahmen (u. a. [4, 5]) bis hin zu gesundheitsabträglichen Effekten, die z. B. mit einer exzessiven Nutzung digitaler Medien verbunden sind. Aktuelle Empfehlungen zur Mediennutzung sehen für Kinder im Alter von 9 bis 12 Jahren die freizeitliche Nutzung von Bildschirmmedien für höchstens 45–60 min am Tag unter Beaufsichtigung vor [6]. In der Altersgruppe der 12- bis 16-Jährigen wird eine tägliche Bildschirmmediennutzungszeit in der Freizeit von 1–2 h empfohlen. Differenzierte Empfehlungen für die Nutzung von sozialen Medien und Videospielen stehen bislang nicht zur Verfügung, jedoch werden riskante oder pathologische Nutzungsmuster zunehmend diskutiert.

Um problematische Nutzungsmuster messbar zu machen, erfolgt in der internationalen Forschungsliteratur oftmals eine Kategorisierung in „normale“, „riskante“ und „problematische“ Mediennutzung. Normale Mediennutzung bezieht sich auf eine Nutzung, die innerhalb der genannten Empfehlungen liegt und nach dem aktuellen Stand der Forschung nicht mit negativen Auswirkungen für die Entwicklung und das tägliche Leben einhergeht. Von riskanter Mediennutzung wird gesprochen, wenn die Nutzung über diese Empfehlungen hinausgeht, jedoch die Wahrscheinlichkeit negativer Konsequenzen nicht substanziell erhöht ist. Problematische Mediennutzung beschreibt Nutzungsmuster, die durch suchtähnliche Symptome gekennzeichnet sind und mit deutlichen Beeinträchtigungen im sozialen, schulischen oder emotionalen Bereich einhergehen [7, 8]. Dabei werden im Kontext sozialer Medien vor allem die kommunikativen und sozialen Aspekte als charakteristische Schlüsselelemente beschrieben, nicht jedoch ein spezifisches Endgerät oder eine Plattform [9].

In Hinblick auf die pathologische Nutzung finden sich neben der Aufnahme der „Gaming Disorder“ in die Internationale Klassifikation der Krankheiten (ICD-11) auch Bemühungen, psychische Störungen im Zusammenhang mit der Nutzung sozialer Netzwerke zu operationalisieren [10, 11]. Unter Einbezug von 63 internationalen Studien konnte in Abhängigkeit von dem jeweils verwendeten Kriterium für 5,0–24,0 % der untersuchten Jugendlichen ein problematisches Nutzungsverhalten von sozialen Medien festgestellt werden [12], während die Prävalenz der Gaming Disorder für Jugendliche in einer neuerlichen Metaanalyse mit 3,3 % angegeben wird [13]. Studien konnten wiederholt nachweisen, dass eine problematische Nutzung sozialer Medien mit einer geringeren Lebenszufriedenheit, häufigeren psychosomatischen Beschwerden, Symptomen von Angst und Depression, Schlafproblemen, einer geringeren körperlichen Aktivität und häufigerem Erleben von Einsamkeit verbunden ist [14–16].

Die deutsche Studienlage ist deutlich begrenzter und weist für fast jeden vierten Heranwachsenden (24,5 %) auf eine extensive Nutzung sozialer Medien und für 11,1 % auf ein riskantes Computerspielverhalten hin [2]. Daten der deutschen „Health Behaviour in School-aged Children“(HBSC)-Studie aus dem Jahr 2013/2014 zeigen zum einen, dass die häufige Nutzung von sozialen Medien mit zunehmendem Alter bei beiden Geschlechtern und bei einem Migrationshintergrund (nur bei Mädchen) ansteigt, während die Nutzung bei Heranwachsenden, die das Gymnasium besuchen, geringer ausgeprägt ist. Zum anderen ließen sich geschlechtsdifferenzierte Zusammenhänge zwischen der häufigen Nutzung sozialer Medien und der subjektiven Gesundheit, der Häufigkeit multipler psychosomatischer Beschwerden sowie der Häufigkeit riskanten Gesundheitsverhaltens (Tabak- und Alkoholkonsum, Rauscherfahrungen) beobachten [17].

Für die Entstehung und Aufrechterhaltung internetbezogener Störungen liegen Modelle vor, die eine Interaktion zwischen relativ stabilen Merkmalen der Person (z. B. Temperament, Motive) und situativ abhängigen affektiven und kognitiven Prozessen (z. B. Belohnungserwartung, spezifischer Coping-Stil) postulieren [18]. Im Kontext problematischer Nutzung sozialer Medien werden zudem verschiedene Richtungshypothesen diskutiert: Die Angst‑/Kompensationshypothese geht davon aus, dass z. B. affektive und soziale Probleme wie Angst, Depression oder Einsamkeit mit einem hohen Bedürfnis nach Zugehörigkeit einhergehen, das in einer intensiven Nutzung sozialer Medien resultiert. Hingegen wird in der belohnungsorientierten Hypothese betont, dass soziale Medien das Bedürfnis nach Selbstdarstellung, Beliebtheit und der Imagepflege bedienen, welches insbesondere bei sozial integrierten und aktiven Personen eine wichtige Rolle bei der Entstehung pathologischer Nutzungsmuster spielen kann [19]. Studienbefunde mit Kindern und Jugendlichen konnten zeigen, dass hohe selbstregulative und kritisch-reflexive Fähigkeiten mit einem geringeren Ausmaß problematischer Mediennutzung verbunden waren, während sich für andere Kompetenzdimensionen (technische Fähigkeit, Interaktionskompetenz) positive Assoziationen zeigten [20, 21].

Auch wenn die Bedeutung von Kompetenzen zunehmend berücksichtigt wird, sind Zusammenhänge zwischen der Nutzung sozialer sowie spielbezogener Medien und der Gesundheitskompetenz (GK) kaum Gegenstand der Forschung. Dies ist insofern erstaunlich, als die GK seit nunmehr etwa 20 Jahren verstärkt in den Fokus der Public-Health-Forschung in Deutschland gerückt ist [22]. Dabei ist die begriffliche und konzeptionelle Heterogenität groß, wobei bestehende Ansätze und Bestimmungsversuche bereits 2012 zu einer übergreifenden Definition verdichtet wurden, die sich in der nationalen und internationalen Forschung und Praxis als diskursbestimmend erwiesen hat. Nach dieser umfasst GK „… das Wissen, die Motivation und die Kompetenzen von Menschen in Bezug darauf, relevante Gesundheitsinformationen in unterschiedlicher Form zu finden, zu verstehen, zu beurteilen und anzuwenden, um im Alltag in den Bereichen der Krankheitsbewältigung, der Krankheitsprävention und der Gesundheitsförderung Urteile fällen und Entscheidungen treffen zu können, die die Lebensqualität im gesamten Lebensverlauf erhalten oder verbessern“ [23].

Während Erwachsene lange Zeit im Fokus der GK-Forschung standen, ist in den letzten 10 Jahren in Deutschland eine Hinwendung zu Kindern und Jugendlichen zu beobachten. Auch hier existieren mittlerweile zahlreiche konzeptionelle Ansätze, in denen GK häufig als individuelles Attribut (kognitiv, affektiv und verhaltensbezogen) verstanden und teilweise im Zusammenspiel sozialer und kontextueller Determinanten betrachtet wird [24]. Das der HBSC-Studie zugrunde liegende Verständnis fasst GK als breites Spektrum an Wissen und Kompetenzen, das junge Menschen in die Lage versetzt, sich selbst, andere und die Welt so zu verstehen, dass sie fundierte Gesundheitsentscheidungen treffen und an den Einflussfaktoren arbeiten können, die ihre eigenen Gesundheitschancen und die der Mitmenschen bestimmen [25].

Ergebnisse der repräsentativen deutschen HBSC-Studie aus dem Jahr 2022 berichten für etwa ein Viertel (24,4 %) der Kinder und Jugendlichen eine geringe GK, mit signifikant ungünstigeren Ausprägungen für 11-Jährige, Heranwachsende mit genderdiverser Geschlechtszugehörigkeit, geringem familiären Wohlstand und jenen, die andere Schulformen als das Gymnasium besuchen [26]. Bestehende Rahmenmodelle postulieren, dass die GK aufgrund eines höheren Gesundheitswissens und der Kenntnisnahme von Strategien zur Beeinflussung von Gesundheit mit einer höheren Wahrscheinlichkeit eines gesundheitsförderlichen Lebensstils einhergeht [23, 27]. Bisher konnten deutsche Studien signifikante Zusammenhänge zwischen einer eingeschränkten GK und dem Ernährungsverhalten, der körperlich-sportlichen Aktivität, dem Tabak- und Alkoholkonsum sowie der Nutzung von Protektoren und Sicherheitsausrüstung zur Unfallprävention empirisch absichern [28–30]. Entsprechend lässt sich annehmen, dass Kinder und Jugendliche mit einer hohen GK die gesundheitlichen Risiken einer exzessiven Mediennutzung besser verstehen und ihr Nutzungsverhalten auf Grundlage ihres Wissens sowie ihrer kritischen Reflexionsfähigkeit angemessen ausrichten können.

Auch wenn es in Deutschland hierzu bisher keine empirischen Befunde gibt, zeigen Ergebnisse der finnischen HBSC-Studie, dass Kinder und Jugendliche mit einer geringen im Vergleich zu einer hohen GK deutlich häufiger ein problematisches Nutzungsverhalten von sozialen Medien aufweisen (22,4 % vs. 9,0 %; [16]). Negative Zusammenhänge ergaben sich in einer Studie aus China vor allem für die Fähigkeit der kritischen Beurteilung, während die funktionale und interaktive GK positiv mit der Internetsucht bei Jugendlichen assoziiert war [31]. Weitere Befunde weisen zudem darauf hin, dass der Zusammenhang zwischen der GK und der problematischen Internetnutzung partiell durch das Wohlbefinden mediiert wird [32].

Vor dem Hintergrund der bestehenden Befundlage zielt der vorliegende Beitrag auf die Untersuchung der Nutzung sozialer und spielbezogener Medien bei Kindern und Jugendlichen in Deutschland. Dabei wird in Anlehnung an Vorbefunde davon ausgegangen, dass Heranwachsende mit geringer GK signifikant häufiger eine problematische Nutzung sozialer Medien und eine intensive Videospielnutzung aufweisen und dass entsprechende Zusammenhänge auch unter Berücksichtigung soziodemografischer und sozioökonomischer Merkmale feststellbar sind.

## Methodik

### Studiendesign und Stichprobe

Es wird auf die aktuellen Daten der „Health Behaviour in School-aged Children“(HBSC)-Studie aus Deutschland zurückgegriffen. Hierbei handelt es sich um die im internationalen Raum größte Studie zum Gesundheitsverhalten und zur Gesundheit von Kindern und Jugendlichen, die 1982 initiiert und mittlerweile in 51 Ländern im Turnus von 4 Jahren nach einheitlichen Standards durchgeführt wird. Deutschland beteiligt sich seit 1993/1994 an dieser Querschnittstudie und adressiert Schülerinnen und Schüler im Alter von 11, 13 und 15 Jahren an allgemeinbildenden Schulen in allen 16 Bundesländern. Mit Ausnahme von Nordrhein-Westfalen (hier entscheidet die Einzelschule über die Beteiligung an Studienvorhaben) wurden in allen Bundesländern Genehmigungsverfahren bei den jeweiligen Kultusministerien durchlaufen.

Die eingeladenen Schulen wurden als Cluster Sample (Klumpenstichprobe) aus der Grundgesamtheit aller Regelschulen in Deutschland gezogen. Um eine repräsentative Schätzung (nahe der Verteilung der Grundgesamtheit) zu erhalten, wurden die Schulgröße sowie die prozentuale Verteilung der Schülerinnen und Schüler stratifiziert nach der Schulform in die Stichprobenziehung eingeschlossen („Probability-Proportional-to-Size“-(PPS-)Design; [33]). Die Befragung in den teilnehmenden Schulen erfolgte im Zeitraum von März bis September 2022 in den Klassenstufen 5, 7 und 9, wahlweise mittels Papier- oder Onlinefragebogen. Die Rekrutierung sah nach der Zufallsauswahl eine schriftliche Einladung der Schulen (postalisch oder per E‑Mail) mit anschließender telefonischer Kontaktaufnahme vor. Zur Incentivierung erhielten alle teilnehmenden Schulen individuelle Teilnahmebescheinigungen sowie eine Zusammenfassung der neuesten Ergebnisse der HBSC-Studie Deutschland.

### Instrumente

Zur Erfassung der problematischen Nutzung von sozialen Medien wurde die Social Media Disorder Scale eingesetzt [34]. Diese umfasst 9 Items mit dichotomer Antwortoption (Ja/Nein), die verschiedene Aspekte eines problematischen Umgangs mit sozialen Medien abbilden (z. B. exzessive Nutzung, sozialer Rückzug, Kontrollverlust, Nutzung zur Emotionsvermeidung) und die sich an den DSM-V-Kriterien der Internet Gaming Disorder orientieren. Die Skala hat sich in vergangenen Studien als valide, reliabel und für den länderübergreifenden Einsatz als geeignet erwiesen [34, 35]. Vergleichbar mit den Ergebnissen vorangegangener Studien erreicht die interne Konsistenz in der deutschen HBSC-Studie zufriedenstellende Werte (α = 0,75). Zur Auswertung und Interpretation der Daten wurde zunächst ein Summenscore für Fälle mit vollständigen Angaben berechnet (Range: 0 bis 9), wobei höhere Werte für einen zunehmend ungünstigen Umgang mit sozialen Medien sprechen. Als Cut-off für ein problematisches Nutzungsverhalten von sozialen Medien gelten Werte ≥ 6, wobei ergänzend Werte von 2 bis 5 als riskante Nutzung und Werte von 0 bis 1 als normale Nutzung definiert wurden [8, 16].

Mittels 2 Einzelitems wurde die Nutzungshäufigkeit digitaler Spiele operationalisiert. Hierbei wurden die Befragten zunächst um eine Angabe der Häufigkeit des Spielens auf einem Smartphone, Tablet, Laptop, PC oder einer Konsole gebeten. Die Antwortoption war 7‑stufig und reichte von „nie/fast nie“ bis „(fast) jeden Tag“. Anschließend wurden die Kinder und Jugendlichen gefragt, wie viel Zeit sie an einem Tag, an dem sie digitale Spiele nutzen, typischerweise damit verbringen. Hierfür standen 5 Antwortkategorien zur Verfügung („1–2 h“ bis „8 h oder mehr“). Für die vorliegende Auswertung wurden die beiden Items zu einer Variable „Spielintensität pro Woche“ zusammengeführt, indem zunächst für die Antwortkategorien einer jeden Variable Mittelwerte gebildet wurden (Häufigkeit der Spielnutzung: 0–7 Tage/Woche, Spieldauer: 1,5–8 h/Spieltag). Anschließend wurde durch Multiplikation eine Annäherung an die wöchentliche Spielintensität in Stunden ermittelt. Für die weiteren Analysen wurden Perzentilwerte entlang von 3 gleich großen Gruppen gebildet: ≤ 3,5 h/Woche, ≤ 9 h/Woche und > 9 h/Woche.

Die Gesundheitskompetenz wurde mithilfe der Skala „Health Literacy for School-Aged Children“ (HLSAC) erfasst [27]. Das Selbstbeurteilungsinstrument umfasst insgesamt 10 Items, deren Zustimmungsgrad auf einer 4‑stufigen Antwortskala bewertet werden konnte („überhaupt nicht zutreffend“ bis „eindeutig zutreffend“). Das Instrument gründet theoretisch auf 5 Komponenten (theoretisches Wissen, praktisches Wissen oder Fähigkeiten, kritisches Denken, Selbstbewusstsein und bürgerschaftliches Denken), die über den Umgang mit Gesundheitsinformation hinausgehen und mit jeweils 2 Items operationalisiert werden. In Vorstudien ergab sich im Rahmen der psychometrischen Testung eine unidimensionale Ausrichtung mit guten bis sehr guten Reliabilitäten [36, 37]. Auch in der vorliegenden Studie ließ sich für die Skala eine hohe interne Konsistenz bestätigen (α = 0,89). Die Auswertung erfolgte entlang von 3 Kategorien, die über den Summenscore (10 bis 40) gebildet wurden, mit folgenden Cut-off-Werten: geringe GK (10 bis 25), moderate GK (26 bis 35) und hohe GK (36 bis 40; [26, 36]).

Als soziodemografische und -ökonomische Merkmale wurden neben dem Geschlecht (männlich, weiblich, genderdivers) die Schulform (Gymnasium vs. andere Schulformen) sowie der Migrationshintergrund und der sozioökonomische Status berücksichtigt. Die Erfassung des Migrationshintergrunds wurde über das eigene sowie das Geburtsland der Mutter und des Vaters erfasst und in 3 Kategorien eingeteilt: kein Migrationshintergrund (Heranwachsende und beide Elternteile in Deutschland geboren), einseitiger Migrationshintergrund (ein Elternteil nicht in Deutschland geboren), zweiseitiger Migrationshintergrund (Heranwachsende selbst und mindestens ein Elternteil oder beide Elternteile nicht in Deutschland geboren; [33]). Der sozioökonomische Status wird in der HBSC-Studie über die Family Affluence Scale (FAS) erhoben. Hierfür wurden die Schülerinnen und Schüler um Auskunft über das Vorhandensein von 6 materiellen Wohlstandsgütern ihres Elternhauses gebeten (Computer, Auto, eigenes Zimmer, Badezimmer, Geschirrspülmaschine, Unternehmen von Urlaubsreisen). Der hierüber ermittelte Summenwert wurde auf Basis einer RIDIT-Kalkulation (Relative to an Identified Distribution Integral Transformation) umgewandelt und in die Gruppen niedriger, mittlerer und hoher familiärer Wohlstand kategorisiert [33].

### Statistische Analysen

Um Aussagen über die Kinder und Jugendlichen in Deutschland in den jeweiligen Altersgruppen treffen zu können, wurden verteilungsbezogene Abweichungen durch eine Gewichtung ausgeglichen, die für die HBSC-Erhebungswelle 2022 erstmals auch die nichtbinäre Erfassung des Geschlechts (genderdivers) berücksichtigte. Alle Analysen wurden unter Verwendung der Gewichtungsvariable durchgeführt, wobei sich die Angaben der absoluten Zahlen auf die ungewichteten Daten beziehen. Zunächst erfolgten univariate Analysen der Daten zur Mediennutzung und der GK über absolute und relative Häufigkeiten. Dem schloss sich die Berechnung von Kreuztabellen mit Chi-Quadrat-Test (χ^2^) an, um bivariate Unterschiede zwischen der Nutzung von sozialen Medien und Spielen nach soziodemografischen und -ökonomischen Merkmalen sowie der GK-Niveaus zu ermitteln. Für die χ^2^-Unabhängigkeitstests wurde ein Signifikanzniveau von *p* < 0,050 festgelegt. Aufgrund der Stichprobengröße wurde Cramérs V als Effektgrößenmaß berechnet und nach Cohen [38] interpretiert: klein (V = 0,1), mittel (V = 0,3) und groß (V = 0,5). Schließlich wurden für die problematische Nutzung sozialer Medien sowie die hohe Spielintensität (> 9 h/Woche) getrennte binärlogistische Regressionen berechnet, um Assoziationen mit den soziodemografischen und -ökonomischen Merkmalen sowie der GK zu ermitteln. Bei den unabhängigen Variablen orientierte sich die Definition der Referenzkategorien an den empirischen Vorbefunden sowie den Ergebnissen der bivariaten Analysen. Die Ergebnisse werden als Odds Ratios (OR) und 95 %-Konfidenzintervall (KI) dargestellt. Signifikante Assoziationen werden entlang der Signifikanzniveaus (*p* < 0,050 bis *p* < 0,001) berichtet. Zur Bewertung der durch die Regressionsmodelle erklärten Varianz wurde Nagelkerkes R^2^ herangezogen [39]. Alle Berechnungen wurden mit der Statistiksoftware IBM SPSS Statistics 29 vorgenommen.

## Ergebnisse

### Beschreibung der Stichprobe und zentraler Untersuchungsmerkmale

An der HBSC-Erhebungswelle 2022 nahmen insgesamt 6475 Schülerinnen und Schüler teil, davon entfallen gewichtet jeweils etwa 49 % auf Jungen und Mädchen, während Heranwachsende mit genderdiverser Geschlechtszuschreibung mit 1,7 % vertreten sind (Tab. 1). Neben einer ausgewogenen Verteilung der Altersgruppen (jeweils 33,3 %) besuchen mehr als ein Drittel der Befragten das Gymnasium, während Gesamtschulen, Realschulen und verbundene Haupt- und Realschulen den größten Anteil der anderen Schulformen ausmachen. Etwas mehr als ein Drittel der Schülerinnen und Schüler weist einen Migrationshintergrund auf, davon 23,1 % einen beidseitigen Migrationshintergrund. Im Hinblick auf den sozioökonomischen Status berichten 17,8 % eine geringe Verfügbarkeit von materiellen Wohlstandsgütern in ihrem Elternhaus.

**Tab. 1:** Beschreibung der Stichprobe und zentraler Untersuchungsmerkmale (*n* = 4862–6475)

	*N*	%
**Geschlecht** (*n* = 6444)		
Jungen	3074	49,2
Mädchen	3258	49,1
Genderdivers	112	1,7
**Alter** (*n* = 6405)		
11 Jahre	2132	33,3
13 Jahre	2160	33,3
15 Jahre	2113	33,3
**Schulform** (*n* = 6475)		
Gymnasium	3393	37,1
Andere	3082	62,9
**Familiärer Wohlstand** (*n* = 6226)		
Hoch	1289	18,9
Mittel	3990	63,3
Niedrig	947	17,8
**Migrationshintergrund** (*n* = 6151)		
Kein	4306	64,8
Einseitig	686	12,1
Beidseitig	1159	23,1
**Gesundheitskompetenz** (*n* = 4862)		
Hoch	774	14,2
Moderat	3007	61,4
Gering	1081	24,4
**Nutzung sozialer Medien** (*n* = 5202)		
Normal	2207	41,5
Riskant	2436	47,4
Problematisch	559	11,1
**Wöchentliche Videospielintensität** (*n* = 5532)		
Bis 3,5 h/Woche	1586	30,2
3,5 bis 9 h/Woche	2116	36,8
> 9 h/Woche	1830	32,9
**Gesamt**	6475	100

Absolute Angaben ungewichtet, prozentuale Angaben gewichtet

Die Ergebnisse der HBSC-Erhebungswelle 2022 zeigen für 24,4 % der Schülerinnen und Schüler eine geringe und für weitere 61,4 % eine moderate GK. Für 11,1 % der Befragten kann ein problematisches Nutzungsverhalten von sozialen Medien ermittelt werden. Entsprechend der statistisch vorgenommenen Einteilung weist ein Drittel der Heranwachsenden eine Spielenutzung von mehr als 9 h in der Woche auf (M = 11,2, SD = 12,61).

### Nutzung unterhaltungsorientierter Medien differenziert nach soziodemografischen, -ökonomischen Merkmalen und Gesundheitskompetenz

Stratifiziert nach Alter und Geschlecht finden sich sowohl für die Nutzung sozialer Medien als auch die Videospielenutzung signifikante Unterschiede (Tab. 2). Während Heranwachsende mit genderdiverser Geschlechtszugehörigkeit (22,4 %) und Mädchen (13,3 %) im Vergleich zu Jungen (8,3 %) deutlich häufiger eine problematische Nutzung sozialer Medien aufweisen, berichten Mädchen im Vergleich zu den beiden anderen Geschlechtsgruppen am seltensten (17,4 % versus 47,8 % bzw. 53,6 %) von einer hohen wöchentlichen Spielintensität (> 9 h). Für die Altersgruppe der 13-Jährigen zeigt sich die höchste Ausprägung sowohl in der problematischen Nutzung sozialer Medien (13,5 %) als auch der hohen wöchentlichen Spielintensität (34,9 %). Schülerinnen und Schüler des Gymnasiums weisen im Vergleich zu Heranwachsenden aus anderen Schulformen seltener problematische Ausprägungen der Mediennutzung auf. Während sich für die Nutzung sozialer Medien keine sozioökonomischen Unterschiede finden lassen, weisen Befragte mit niedrigem im Vergleich zu jenen mit hohem familiären Wohlstand häufiger eine hohe Spielintensität (> 9 h/Woche) auf (35,5 % versus 27,2 %). Schließlich ergeben sich für die problematische Nutzung sozialer Medien Unterschiede zuungunsten von Kindern und Jugendlichen mit (insbesondere beidseitigem) Migrationshintergrund, während sich für die wöchentliche Spielintensität signifikante, jedoch weniger deutliche Unterschiede zuungunsten von Heranwachsenden ohne Migrationshintergrund ergeben. Werden die Effektgrößen nach Cramérs V herangezogen, so fallen die meisten signifikanten Unterschiede unter die Schwelle kleiner Effekte. Lediglich die geschlechts-, alters- und schulformbezogenen Unterschiede der wöchentlichen Spielintensität weisen auf kleine Effektgrößen (V ≥ 0,1) hin; die für das Geschlecht liegen an der Schwelle zu mittleren Effekten.

**Tab. 2:** Nutzung sozialer Medien und Videospielintensität differenziert nach soziodemografischen und -ökonomischen Merkmalen

	Nutzung sozialer Medien			Videospielintensität		
	Normal % (*n*)	Riskant % (*n*)	Problematisch % (*n*)	≤ 3,5 h/Woche % (*n*)	≤ 9 h/Woche % (*n*)	> 9 h/Woche % (*n*)
**Geschlecht**	*χ2 (df* *=* *4)* *=* *91,63, p* *<* *0,01, V* *=* *0,09*			*χ2 (df* *=* *4)* *=* *884,73, p* *<* *0,01, V* *=* *0,28*		
Jungen	47,4 % (1168)	44,3 % (1091)	8,3 % (204)	13,9 % (375)	38,4 % (1037)	47,8 % (1291)
Mädchen	36,5 % (950)	50,2 % (1306)	13,3 % (346)	46,8 % (1264)	35,8 % (969)	17,4 % (470)
Genderdivers	26,5 % (26)	51,1 % (50)	22,4 % (22)	25,8 % (25)	20,6 % (20)	53,6 % (52)
**Alter**	*χ2 (df* *=* *4)* *=* *59,89, p* *<* *0,01, V* *=* *0,08*			*χ2 (df* *=* *4)* *=* *143,43, p* *<* *0,01, V* *=* *0,11*		
11 Jahre	48,4 % (741)	41,3 % (632)	10,3 % (158)	23,3 % (403)	47,1 % (813)	29,5 % (510)
13 Jahre	38,3 % (676)	48,2 % (850)	13,5 % (239)	30,0 % (560)	35,2 % (657)	34,9 % (652)
15 Jahre	38,9 % (726)	51,7 % (965)	9,4 % (175)	36,7 % (700)	29,2 % (557)	34,1 % (650)
**Schulform**	*χ2 (df* *=* *2)* *=* *33,29, p* *<* *0,01, V* *=* *0,08*			*χ2 (df* *=* *2)* *=* *48,40, p* *<* *0,01, V* *=* *0,12*		
Gymnasium	46,2 % (909)	44,7 % (878)	9,1 % (179)	29,5 % (605)	43,6 % (895)	26,9 % (551)
Andere	38,6 % (1234)	49,1 % (1569)	12,3 % (3196)	30,7 % (1059)	32,8 % (1131)	36,5 % (1261)
**Familiärer Wohlstand**	*χ2 (df* *=* *4)* *=* *7,86, n.* *s.*			*χ2 (df* *=* *4)* *=* *53,95, p* *<* *0,01, V* *=* *0,07*		
Hoch	42,0 % (410)	46,3 % (453)	11,7 % (114)	32,4 % (330)	40,5 % (413)	27,2 % (277)
Mittel	42,1 % (1352)	47,5 % (1524)	10,4 % (332)	28,3 % (960)	37,8 % (1285)	33,9 % (1153)
Niedrig	38,7 % (343)	47,7 % (423)	13,6 % (121)	36,2 % (351)	28,4 % (275)	35,5 % (344)
**Migrationshintergrund**	*χ2 (df* *=* *4)* *=* *59,38, p* *<* *0,01, V* *=* *0,08*			*χ2 (df* *=* *4)* *=* *32,34, p* *<* *0,01, V* *=* *0,06*		
Kein	45,1 % (1448)	45,6 % (1463)	9,3 % (298)	27,7 % (935)	39,0 % (1317)	33,3 % (1125)
Einseitig	35,8 % (216)	50,8 % (307)	13,4 % (81)	31,9 % (204)	36,8 % (236)	31,3 % (200)
Beidseitig	34,3 % (379)	51,3 % (567)	14,4 % (159)	35,1 % (420)	31,2 % (374)	33,7 % (403)

*n* Häufigkeit, *χ*^*2*^ Chi Quadrat, *n.* *s.* nicht signifikant, *V* Cramérs V

**Abb. 1:**
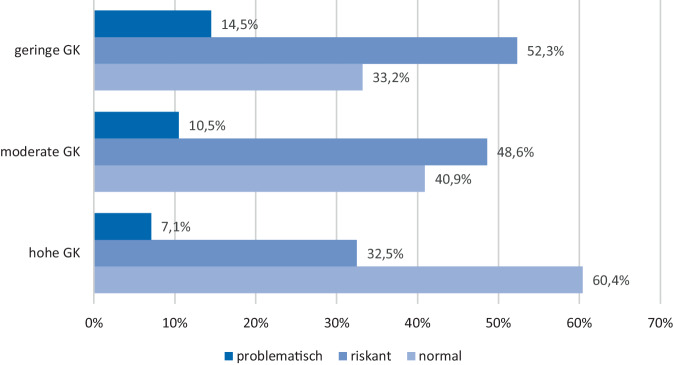
Nutzung sozialer Medien stratifiziert nach Gesundheitskompetenz (*GK*) bei Schülerinnen und Schülern (*N* = 4510), *χ2* *(df* *=* *4)* *=* *129,71, p* *<* *0,001, V* *=* *0,12. *Quelle: eigene Abbildung

Die Ergebnisse zu Unterschieden in der Mediennutzung nach der GK sind in den Abb. 1 und 2 dargestellt. Erwartungskonform zeigt sich, dass Schülerinnen und Schüler mit geringer GK im Vergleich zu Heranwachsenden mit hoher GK doppelt so häufig ein problematisches Nutzungsverhalten von sozialen Medien aufweisen (14,5 % versus 7,1 %). Ähnliche Befunde gibt es für die Spielenutzung. Hier beträgt die Differenz derjenigen, die wöchentlich mehr als 9 h digitale Spiele nutzen zwischen Heranwachsenden mit geringer oder hoher GK mehr als 12,0 %. Die über Cramérs V ermittelten Effektgrößen liegen auch hier unterhalb der Schwelle oder im Rahmen kleiner Effekte.

**Abb. 2:**
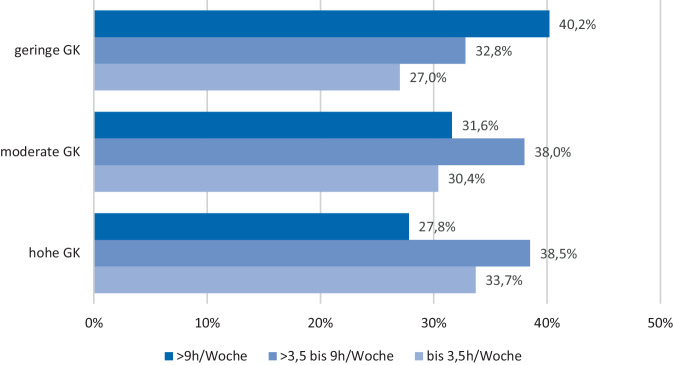
Wöchentliche Videospielintensität stratifiziert nach Gesundheitskompetenz (*GK*) bei Schülerinnen und Schülern (*N* = 4663), *χ2* *(df* *=* *4)* *=* *37,85, p* *<* *0,001, V* *=* *0,06. *Quelle: eigene Abbildung

### Multivariate Vorhersage der Nutzung unterhaltungsorientierter Medien

Die Ergebnisse der Regressionsanalysen zeigen für die problematische Mediennutzung und die hohe Spielintensität partiell unterschiedliche Zusammenhangsmuster mit den soziodemografischen und -ökonomischen Merkmalen sowie der GK (Tab. 3). So geht die Zugehörigkeit zu den Geschlechtskategorien weiblich und genderdivers im Vergleich zu Jungen mit einer bis über 3‑fach erhöhten Wahrscheinlichkeit (OR = 1,89–3,25, *p* < 0,001) einer problematischen Nutzung sozialer Medien einher, während Mädchen im Vergleich zur Referenzgruppe der Jungen eine signifikant geringere Wahrscheinlichkeit (OR = 0,24, *p* < 0,001) einer hohen Spielintensität aufweisen. Wie in den bivariaten Ergebnissen ist die Zugehörigkeit zur Altersgruppe der 13-Jährigen im Vergleich zu den 11-Jährigen sowie zu einer anderen Schulform als das Gymnasium mit einer erhöhten Nutzung beider Medienformen verbunden. Ein geringer familiärer Wohlstand erweist sich nur für die hohe Spielintensität als prädiktiv (OR = 1,27, *p* < 0,05), während ein Migrationshintergrund mit einer etwa 1,5-fach erhöhten Wahrscheinlichkeit einer problematischen Mediennutzung verbunden ist.

**Tab. 3:** Auftretenswahrscheinlichkeit einer problematischen Nutzung von sozialen Medien (*n* = 4277) und einer hohen Videospielintensität (*n* = 4381), multivariates logistisches Regressionsmodell mit Einschluss aller Prädiktoren

	Problematische Nutzung sozialer Medien		Hohe Videospielintensität (> 9 h/Woche)	
	OR	95 %-KI	OR	(95 %-KI)
**Geschlecht**				
Jungen (Ref.)	1,00	–	1,00	–
Mädchen	1,89	1,54–2,33**	0,24	0,21–0,27**
Genderdivers	3,25	1,82–5,80**	0,96	0,60–1,54
**Alter**				
11 Jahre (Ref.)	1,00	–	1,00	–
13 Jahre	1,39	1,08–1,79*	1,36	1,15–1,62**
15 Jahre	0,88	0,68–1,14	1,25	1,06–1,49*
**Schulform**				
Gymnasium (Ref.)	1,00	–	1,00	–
Andere	1,25	1,00–1,56*	1,41	1,22–1,63*
**Familiärer Wohlstand**				
Hoch (Ref.)	1,00	–	1,00	–
Mittel	0,83	0,64–1,08	1,16	0,97–1,40
Niedrig	1,06	0,77–1,46	1,26	1,00–1,59*
**Migrationshintergrund**				
Kein (Ref.)	1,00	–	1,00	–
Einseitig	1,51	1,13–2,02*	0,82	0,66–1,02
Zweiseitig	1,50	1,19–1,89**	0,84	0,71–0,99
**Gesundheitskompetenz**				
Hoch (Ref.)	1,00	–	1,00	–
Moderat	1,39	0,98–1,95	1,18	0,96–1,45
Gering	2,04	1,41–2,94**	1,63	1,30–2,06**
**Nagelkerkes R**^**2**^	0,053		0,161	

*OR* Odds Ratio, *KI* Konfidenzintervall, *Ref.* Referenzgruppe

*p < 0,05; **p < 0,001

Schließlich weisen Schülerinnen und Schüler mit einer geringen im Vergleich zu einer hohen GK eine signifikant höhere Wahrscheinlichkeit einer problematischen Nutzung sozialer Medien und einer hohen Spielintensität auf (OR = 1,63–2,04, *p* > 0,001). Insgesamt beträgt der über die Regressionsmodelle erklärte Teil der Varianz in der problematischen Nutzung sozialer Medien 5,3 % und für die hohe Spielintensität 16,1 %.

## Diskussion

Mit dem vorliegenden Beitrag werden erstmals Daten zum Zusammenhang zwischen problematischen Formen der Mediennutzung und der GK bei Kindern und Jugendlichen in Deutschland vorgelegt. Damit werden bisherige Befunde erweitert, die bereits Assoziationen zwischen der GK und anderen Formen des Gesundheitsverhaltens (z. B. körperliche Aktivität, Ernährungsverhalten, Substanzkonsum) feststellen konnten [28–30]. Die Ergebnisse der aktuellen HBSC-Studie Deutschland zeigen, dass 11,1 % der 11-, 13- und 15-jährigen Schülerinnen und Schüler eine problematische Nutzung sozialer Medien mit suchtähnlichen Mustern aufweisen und etwa ein Drittel mehr als 9 h in der Woche mit digitalen Spielen verbringt. Die multivariaten Analysen zeigen eine höhere Wahrscheinlichkeit problematischer Nutzung sozialer Medien für Mädchen und Heranwachsende mit genderdiverser Geschlechtszugehörigkeit, für 13-Jährige sowie für Befragte mit Migrationshintergrund und jene, die eine andere Schulform als das Gymnasium besuchen. Hingegen ist die hohe Spielintensität vor allem bei Jungen und Heranwachsenden mit genderdiverser Geschlechtszuschreibung, 13- und 15-Jährigen sowie bei Befragten mit geringem familiären Wohlstand zu beobachten. Darüber hinaus steht eine geringe GK sowohl mit der Nutzung sozialer Medien als auch einer hohen Spielintensität in Verbindung, wobei Kinder und Jugendliche mit einer hohen GK seltener eine problematische Nutzung von sozialen Medien und seltener eine hohe Spielintensität zeigen.

Damit können Befunde der finnischen HBSC-Studie auch für Kinder und Jugendliche aus Deutschland bestätigt werden [16]. Für die Erklärung dieser Zusammenhänge sind direkte wie auch indirekte Argumentationslinien denkbar. Wie bereits ausgeführt, kann auf Basis vorhandener GK-Rahmenmodelle [23, 27] davon ausgegangen werden, dass Heranwachsende mit hoher GK über ein höheres Gesundheitswissen und eine höhere kritische Reflexionsfähigkeit verfügen, die es ihnen ermöglichen, das eigene Nutzungsverhalten vor dem Hintergrund potenzieller gesundheitlicher Konsequenzen auszurichten. Hier zeigen sich auch Verbindungen zwischen dem Konstrukt der GK und den bereits in Vorstudien untersuchten Zusammenhängen zwischen selbstregulativen und kritisch-reflexiven Fähigkeiten sowie der problematischen Mediennutzung [20, 21]. Gleichzeitig ist es auch möglich, dass Heranwachsende im Onlineraum auf hilfreiche Gesundheitsinformationen treffen [4, 5], sodass die GK gestärkt wird. Dies kann sich wiederum positiv auf das Nutzungsverhalten von digitalen Medien auswirken, sodass hier eine komplexe Interaktion mit der GK entsteht. Denkbar ist auch, dass Eltern von Kindern und Jugendlichen mit hoher GK das Mediennutzungsverhalten stärker regulieren oder häufiger Anreize für nichtdigitale Freizeitaktivitäten setzen. Tatsächlich ließen sich zwischen der elterlichen Unterstützung und Aufsicht sowie der Nutzung sozialer Medien in Analysen von HBSC-Daten aus anderen Ländern signifikante Zusammenhänge mit der sozialen Mediennutzung absichern [40]. Hierbei kommt auch dem familiären Wohlstand als Indikator des sozioökonomischen Status eine bedeutsame Rolle zu, der positiv mit der GK assoziiert ist [26]. Kinder und Jugendliche mit geringem familiären Wohlstand weisen nicht nur häufiger eine geringe GK auf, sondern zeigen aufgrund der geringen GK auch häufiger eine problematische Mediennutzung, was sie in mehrfacher Hinsicht als Gruppe mit erhöhter Vulnerabilität kennzeichnet.

Schließlich lässt sich ein weiterer indirekter Erklärungspfad anführen, der an den Motiven der (exzessiven) Mediennutzung ansetzt. So konnten Studien in der Vergangenheit herausstellen, dass Stress, negative Emotionen und soziale Probleme sowie deren Bewältigung (z. B. in Form von Eskapismus) u. a. infolge eines gesteigerten Bedürfnisses nach Zugehörigkeit mit der problematischen Medien- und Spielnutzung verbunden sind [19, 41, 42]. Ein in vorangegangenen Studien zugrunde gelegtes GK-Rahmenmodell geht davon aus, dass Bewältigungsfähigkeiten oder Kontrollüberzeugungen die Beziehung zwischen der GK und Indikatoren der Gesundheit mediieren können [43]. Auch wenn die konzeptionelle Verknüpfung empirisch noch nicht abgesichert ist, verfügen Heranwachsende mit hoher GK wahrscheinlich über ein größeres Repertoire der adaptiven Regulation und Bewältigung von Anforderungen, wodurch sie seltener auf digitale Medien zurückgreifen. Dieser Erklärungspfad könnte insbesondere für Heranwachsende mit genderdiverser Geschlechtszuschreibung von besonderer Relevanz sein, die im Vergleich zu Jungen und Mädchen deutlich häufiger eine hohe bzw. problematische Nutzung digitaler Medien aufweisen. Dabei berichten Jugendliche, die sich als transgender und genderdivers identifizieren, das Internet und soziale Medien häufig zum Experimentieren mit ihrer Geschlechtsidentität zu nutzen [44], was auf einen großen Orientierungs- und Informationsbedarf hindeutet, der sie auch anfälliger für gesundheitliche Fehlinformationen machen könnte. Eine aktuelle Übersichtsarbeit konnte zudem herausarbeiten, dass Videospiele für transgender und genderdiverse Jugendliche eine Möglichkeit der Exploration und Entfaltung (z. B. über gestaltbare Avatare), aber auch der Alltagsflucht zur Bewältigung von Stress sowie eine Quelle sozialer Kontakte und Unterstützung darstellen [45], was die höhere Spielintensität in dieser Gruppe erklären könnte. Auch wenn infolge der geringen Fallzahlen eine vorsichtige Interpretation angeraten ist, deuten diese Ergebnisse einen besonderen Präventionsbedarf für diese Gruppe an, nicht zuletzt auch deshalb, weil der Anteil derjenigen mit geringer GK unter den Heranwachsenden mit genderdiverser Geschlechtszuschreibung mit 51,0 % besonders hoch ausfällt [26]. Daraus folgt insbesondere auch die Notwendigkeit zur Schaffung besserer Rahmenbedingungen für genderdiverse Heranwachsende außerhalb des Onlineraumes, damit sie auch jenseits der digitalen Welt soziale Kontakte und Unterstützung sowie Explorationsmöglichkeiten finden und nutzen können.

Neben den bereits berichteten Assoziationen zwischen dem familiären Wohlstand und der hohen Spielintensität ließen sich für den Migrationshintergrund lediglich Zusammenhänge mit der problematischen Nutzung sozialer Medien absichern. Dies zeigte sich bereits in vorangegangenen HBSC-Befunden aus Luxemburg [40] und könnte dadurch erklärt werden, dass Kinder und Jugendliche mit Migrationshintergrund soziale Medien nutzen, um mit ihrem Herkunftsland oder dort verbliebenen Familienmitgliedern oder Freunden in Kontakt zu bleiben.

### Stärken und Limitationen

Mit diesem Beitrag werden erstmals Daten zum Zusammenhang zwischen problematischer Mediennutzung und GK bei Kindern und Jugendlichen in Deutschland vorgestellt, die aufgrund der Repräsentativität eine hohe Aussagekraft für die Altersgruppe der 11-, 13- und 15-Jährigen haben. Weitere Stärken bestehen im Einsatz validierter Instrumente, in der hohen methodischen Standardisierung sowie in der Möglichkeit des länderübergreifenden Vergleichs von Daten infolge der internationalen Studienausrichtung.

Die HBSC-Studie wird zwar im Turnus von 4 Jahren durchgeführt, dennoch sind kausale Rückschlüsse aufgrund des querschnittlichen Studiendesigns nicht möglich. Auch wenn GK aufgrund theoretischer Modelle und Überlegungen in den vorliegenden Analysen als Prädiktor für das Nutzungsverhalten von digitalen Medien betrachtet wird, sind in der Realität komplexe Interaktionen anzunehmen, die in künftigen Studien und Analysen zu untersuchen sind. Im Hinblick auf den Befragungszeitpunkt ab März 2022 ist das damalige durch die Omikron-Welle gekennzeichnete COVID-19-Pandemiegeschehen zu berücksichtigen. Zwar waren die Infektionszahlen nach einem jahresanfänglichen Anstieg ab April rückläufig, sie könnten aber infolge von Restriktionen zu einer erhöhten Mediennutzung bei Kindern und Jugendlichen geführt haben. Bei der Interpretation der Ergebnisse ist zudem zu bedenken, dass die für die Datenanalysen gebildete Variable „Videospielintensität“ lediglich eine sehr grobe quantitative Annäherung an das problematische Spielverhalten darstellt. So unterliegt die für beide Spielvariablen vorgenommene Mittelwertbildung der Antwortkategorien einer geringen Genauigkeit, weshalb künftige Studien auch für diesen Bereich auf elaborierte Instrumente der dysfunktionalen und pathologischen Nutzung zurückgreifen sollten (z. B. [10, 46]). Darüber hinaus sollte bei der Bewertung der Ergebnisse bedacht werden, dass die Einteilung in „normale“, „riskante“ und „problematische“ Mediennutzung auf etablierten Skalen basiert, die häufig normative Annahmen über einen gesunden oder angemessenen Medienkonsum zugrunde legen. Diese Kategorien spiegeln jedoch nicht zwangsläufig universelle Normen wider, sondern können stark von sozialen, kulturellen und individuellen Kontexten abhängen. Zukünftige Forschung sollte daher stärker auf die Kontextabhängigkeit von Mediennutzungsverhalten ausgerichtet werden. Weiterhin ist zu berücksichtigen, dass die GK, wie in der überwiegenden Mehrheit der empirischen Forschung, in Form subjektiver Fähigkeitsurteile erfasst wurde, die immer auch mit Problemen von Fehleinschätzungen verbunden sind. Gerade bei jüngeren Altersgruppen werden infolge der Komplexität der Items Schwierigkeiten der Beantwortung vermutet, die zu Antwortverzerrungen und fehlenden Werten führen können [37]. Schließlich ist der über die Regressionsmodelle erklärte Anteil an Varianz in den abhängigen Variablen (insbesondere für die problematische Nutzung sozialer Medien) gering und legt den Einbezug weiterer Prädiktoren nah. Neben den in der Diskussion genannten Faktoren erwies sich die Einsamkeit für die Erklärung der Mediennutzung relevant und weitergehende Analysen liefern Indizien dafür, dass die GK den Zusammenhang zwischen der problematischen Nutzung sozialer Medien und der Gesundheit moderieren kann [16, 47].

### Fazit und Implikationen

Die Befunde zeigen einen Handlungsbedarf, der sich aus einem nicht unbeträchtlichen Anteil der 11-, 13- und 15-Jährigen in Deutschland mit einem problematischen digitalen Medienumgang ergibt. Zudem erlauben die in dieser Studie ermittelten Zusammenhänge mit soziodemografischen und sozioökonomischen Merkmalen die Identifikation von Personen mit besonderem Präventionsbedarf. Kürzlich veröffentlichte Empfehlungen schlagen 3 Gruppen von Public-Health-Interventionen vor [48]: Neben Strategien der Hinauszögerung der Nutzung digitaler Medien und Geräte (z. B. durch Altersbegrenzungen, Warnhinweise auf Geräteverpackungen oder Kampagnen zur Sensibilisierung und Verhaltensänderung) werden Maßnahmen zur Reduktion der Medien- und Gerätenutzung, wie z. B. Produktsteuer, bessere Nutzung bestehender technischer Features (z. B. Nutzungsstatistik, automatische Zeitbegrenzung und Notifications) oder Schaffung von gerätefreien Räumen, aufgeführt. Schließlich wird die Verringerung der schädlichen Folgen der Nutzung digitaler Medien und Geräte als dritte Strategie genannt, wobei der Stärkung von allgemeiner und digitaler GK eine hohe Bedeutung zugesprochen wird. Die Schule stellt hierbei ein zentrales Setting dar, da sie entsprechend ihres Bildungs- und Erziehungsauftrags für die Vermittlung von Medienkompetenzen verantwortlich ist. Vor dem Hintergrund der Passung der in den Bundesländern vorliegenden Medienkompetenzrahmen mit den Dimensionen der GK besteht die Chance der reibungsarmen Integration des Themas Gesundheit in die Schule [49].
