# Erratum zu: Problematische Nutzung digitaler Medien und Gesundheitskompetenz von Schülerinnen und Schülern in Deutschland. Befunde der HBSC-Studie 2022

**DOI:** 10.1007/s00103-025-04034-4

**Published:** 2025-03-20

**Authors:** Kevin Dadaczynski, Anne Kaman, Ulrike Ravens-Sieberer, Saskia M. Fischer, Ludwig Bilz, Saskia Sendatzki, Ronja M. Helmchen, Katharina Rathmann, Matthias Richter, HBSC-Studienverbund Deutschland

**Affiliations:** 1 Fachbereich Gesundheitswissenschaften, Hochschule Fulda, Leipziger Straße 123, 36037 Fulda, Deutschland; 2 Zentrum für Angewandte Gesundheitswissenschaften, Leuphana Universität Lüneburg, Lüneburg, Deutschland; 3 Zentrum für Psychosoziale Medizin, Klinik für Kinder- und Jugendpsychiatrie, -psychotherapie und -psychosomatik, Forschungssektion Child Public Health, Universitätsklinikum Hamburg-Eppendorf, Hamburg, Deutschland; 4 Fakultät für Humanwissenschaften, Brandenburgische Technische Universität Cottbus-Senftenberg, Senftenberg, Deutschland; 5 Fachbereich Sozialwesen, Hochschule RheinMain, Wiesbaden, Deutschland; 6 School of Medicine and Health, Lehrstuhl Social Determinants of Health, Technische Universität München, München, Deutschland; 7 Public Health Zentrum Fulda (PHZF), Hochschule Fulda, Fulda, Deutschland

**Erratum zu:**

**Bundesgesundheitsbl 2025**

10.1007/s00103-025-04008-6

In diesem Artikel fehlte „HBSC-Studienverbund Deutschland“ in der Autorenliste und die folgenden Informationen zum HBSC-Studienverbund wurden am Ende des Artikels eingefügt:

## Mitglieder des HBSC-Studienverbund Deutschland.

Der HBSC-Studienverbund Deutschland setzt sich aktuell aus den folgenden Standorten zusammen: Brandenburgische Technische Universität Cottbus-Senftenberg (Prof. Dr. Ludwig Bilz), Pädagogische Hochschule Heidelberg (Prof. Dr. Jens Bucksch), Hochschule Fulda (Prof. Dr. Katharina Rathmann, Prof. Dr. Kevin Dadaczynski – nationale Co-Leitung), Martin-Luther-Universität Halle-Wittenberg (Dr. Irene Moor – nationale Co-Leitung), Technische Universität München (Prof. Dr. Matthias Richter), Universität Tübingen (Prof. Dr. Gorden Sudeck) und Universitätsklinikum Hamburg-Eppendorf (Prof. Dr. Ulrike Ravens-Sieberer).

Der Originalbeitrag wurde korrigiert.

